# Annotations

**Published:** 1895-10-12

**Authors:** 


### Annotations.

### The Dangers of Street Cycling.

Miss Eleanor Ormerod, the well-known entomologist, gives an account in a contemporary of the way in which the late Professor C. V. Riley met his death while cycling. Professor Riley left his home at Wyoming Avenue, Washington, on his bicycle, and rode in the direction of the city. He seems to have been riding at great speed; and at a point in the road where two streets intersect each other, he came in contact with an obstacle which checked the bicycle, and threw the rider violently forward over the handle-bars. The professor fell with great force on his head and face, and was picked up unconscious, and taken home, where he died in a few hours. Consciousness never returned. At the post-mortem examination it was found that in addition to superficial injuries about the head and face, he had fractured the base of the skull. This is by no means the first serious accident which has resulted from riding a bicycle at a high rate of speed in the public streets. Not long ago the writer saw a young man thrown over the handles of his bicycle under similar circumstances, and it was feared for a while that he had been killed. Cyclists, especially youthful ones, have been in the habit of riding quickly, both in streets and lanes, purely for their own pleasure; and when warned that the public are thereby subjected to great risks, it has been their pleasure to reply that "the public must take care of itself." It begins to be evident that fast street riding may be almost as dangerous to cyclists themselves as to the public. It is in fact extremely dangerous to both parties. Not more than a fortnight or three weeks ago an aged man was knocked down by a youthful cyclist at Crowborough, and died in a few hours. Is it not time that the legislature should interfere, and regulate the rate of speed at which cyclists may ride their machines, at any rate in or near the streets of villages and towns?

### Relieving Officers and Medical Extras.

A question of very great importance in regard to the administration of the poor law has been raised by a correspondent in the *Lancet*, who calls attention to the fact that relieving officers sometimes pay no attention to the recommendations given by the doctor for medical extras. A case is related in which a patient was ordered linseed meal for poulticing and beef with which to make beef-tea. The linseed was given, but the beef was withheld. In another case, beef and brandy were ordered. Here the beef was given, but the brandy was withheld. But details are unnecessary; the question is one of principle. Is the relieving officer to review the treatment ordered by the doctor? We need have no hesitation in stating the case in this form, for the treatment of acute disease is becoming every year more and more a matter of nursing and careful diet. Critical points and awkward corners occur in the course of these maladies when drugs are absolutely life-saving, but the exhibition of drugs in an unfed patient is like the scattering of seed on an untilled and barren soil. At this time of day it is ridiculous to separate food from medicine, or for a relieving officer to undertake to decide how far the doctor shall go in the treatment of his patient. It seems to be true, unfortunately, that the so-called order for extra diet, given by the medical officer, is but a recommendation, and has no compelling power; and at the meeting of the Board of Guardians at which the matter was discussed, the opinion was expressed that the relieving officer was quite within his rights in refusing to honour this order if he thought fit. It does not seem, however, to have been stated that in acting thus he does so at his peril. The *Lancet* says: "It ought to be widely known that a relieving officer has *no* absolute discretion in dealing with a case of urgent necessity, and, therefore, if he refuses to carry out the medical recommendation for food and stimulants, and the patient dies, he can be held responsible"; and quotes a case in which Mr. Justice Hawkins made some very severe remarks on the defendant relieving officer, whose conviction was affirmed. As bearing on this matter, we would refer to a recommendation, contained in a circular of the Poor Law Board so far back as 1868, to the effect "That as the recommendations of medical officers for meat and stimulants are regarded as equivalent to orders for additional relief, they should in all cases be accompanied by a report from the medical officer in a prescribed form, setting forth the particulars of each case, ascertained by personal inquiry." In view, then, of the fact that this circular definitely states that the doctor's recommendations "are regarded as equivalent to orders for additional relief," it behoves relieving officers to exercise with great judgment such discretion as they may legally possess.

### Penny Bottles of Sterilised Milk.

It must be understood that the substance which goes by the name of sterilised milk is something different from ordinary milk. The albumen is converted into peptone, and the relation of the fat to the other constituents is in some way changed, so that very little fat separates from the emulsion. From the investigations, however, made by Dr. Blasius, Professor of Hygiene at Brunswick, it appears that the nutritive properties of the milk are in no way interfered with, and that the practitioners in the town had expressed themselves as highly satisfied with the results of its use in the cases in which they had ordered it. The business of sterilization is evidently carried on at Brunswick on a large scale, which probably accounts for its successful issue. The milk is placed in bottles, containing each one-third of a litre, and submitted to steam at a temperature of 103 deg. C., the bottles being all the time agitated to prevent separation. These bottles, each containing a third of a litre, are sold for a penny a-piece. This does not strike one as cheap, but the advantages of obtaining milk in a form in which it will not go bad, which obviates the necessity of making the day's consumption fit the day's supply, and above all the advantage of knowing that the milk is safe without any further boiling, are so great that it must surprise those who understand the importance of these things that sterilised milk has not come into far greater favour than has been the case.

